# Assessing the potential repercussions of the COVID-19 pandemic on global SDG attainment

**DOI:** 10.1007/s43621-021-00067-2

**Published:** 2022-01-18

**Authors:** Hideyuki Doi, Takeshi Osawa, Narumasa Tsutsumida

**Affiliations:** 1 Graduate School of Information Science, University of Hyogo, 7-1-28 Minatojima-minamimachi, Chuo-ku, Kobe, 650-0047 Japan; 2 Graduate School of Urban Environmental Sciences, Tokyo Metropolitan University, Minami-Osawa 1-1, Hachiouji, Tokyo 192-0397 Japan; 3 Graduate School of Science & Engineering, Saitama University, 255 Shimo-Okubo, Sakura ward, Saitama, Saitama 338-8570 Japan

**Keywords:** SDGs, COVID-19, Human movement, NO_2_ emissions

## Abstract

The coronavirus disease (COVID-19) pandemic has led to a worldwide lockdown, and this restriction on human movements and activities has significantly affected society and the environment. Some effects might be quantitative, but some might be qualitative, and some effects could prolong immediately and/or persistently. This study examined the consequences of global lockdown for human movement and nitrogen dioxide (NO_2_) emissions using an air pollution index and dataset and satellite image analyses. We also evaluated the immediate (during lockdown) and persistent (after lockdown) effects of lockdown on achieving the SDGs. Our analysis revealed a drastic reduction in human movement and NO_2_ emissions and showed that many SDGs were influenced both immediately and persistently due to the global lockdown. We observed the immediate negative impacts on four goals and positive impacts on five goals, especially those concerning economic issues and ecosystem conservation, respectively. The persistent effects of lockdown were likely to be predominantly reversed from their immediate impacts due to economic recovery. The global lockdown has influenced the global community’s ability to meet the SDGs, and our analysis provides powerful insights into the status of the internationally agreed-upon SDGs both during and after the COVID-19-induced global lockdown.

**Supplementary Information:**

The online version contains supplementary material available at 10.1007/s43621-021-00067-2.

## Introduction

Society changed drastically in 2020 due to the coronavirus disease (COVID-19) pandemic. COVID-19 was first observed in Wuhan, China, and had spread to every continent by April 2020 [1, 2]. To reduce the spread of COVID-19, China imposed a lockdown in Wuhan City on 23 January 2020 [1]. WHO reported 202,138,110 infected cases, with 4,285,299 confirmed deaths in 215 countries and territories around the world resulting from COVID-19 up to 8 August 2021 (URL: https://www.who.int/publications/m/item/weekly-operational-update-on-covid-19---9-august-2021). The disease has caused a massive global health challenge and created ripples in the medical fraternity [1, 2]. Undoubtedly, unprecedented strategies are required, such as the massive surveillance to prevent spreading and the creation of a sophisticated network of diagnostics and medical facilities for immediate detection and treatment of the disease.

A major implication of the pandemic were ‘lockdowns’ both at the global and local scales since by April 2020, people in many countries were under strict movement restrictions [1]. Besides restricting movement, lockdowns also affected educational, political, and economic activities [3], with the resulting consequences expected to significantly impact both society and the environment [4–7]. Significant effects of the lockdown have already been observed on the global economy [8], air pollution [9–11], and wildlife conservation [5]. Here, multiple levels of lockdown policies [12] are considered that restrict society and behaviour. Also, the significant effects were recovered after reducing the lockdown, especially economic recovery occurred in both lower-middle-income countries and high-income countries [5].

However, the effects of various global lockdown restrictions on society and the environment have not been sufficiently evaluated and synthesised as we introduced above, especially the resulting environmental footprint. Herein, we summarise the consequences of the global lockdown on society and the environment using air pollution and human movement indices, particularly focusing on the environmental footprint. Using case studies and predictions related to the COVID-19-induced global lockdown, we evaluate and debate the impacts of the global lockdown on current and future sustainable development comprehensively.

The roadmap with goals and indicators for the sustainable development of human society was established by the United Nations as the Sustainable Development Goals (SDGs) [13]. The SDGs set an agenda for 2030 to transform the world by simultaneously ensuring human well-being, economic prosperity, and environmental conservation [13]. The SDGs serve as milestones to pave the way for sustainable development for both developing and developed countries. The SDGs, comprising 17 goals and 169 targets, address the challenges faced by humanity. With their corresponding targets, they expand upon various aspects of sustainable development, including societal structure, economy, policy, and sustainable ecosystem use [13, 14]. Some studies have suggested that COVID-19 can affect SDG achievements [4, 6, 7], but these studies have not evaluated the effect of the pandemic on all SDG targets. We focus on all SDG achievement as a proxy to evaluate global lockdown impacts on current and future sustainable development. Although that approach is qualitative, we could evaluate the effects of global lockdown impacts comprehensively. Current global responses to the COVID-19 crisis are likely to impact the ability to deliver all SDGs within the intended timescales thereby leading to uncertainties [15].

**Fig. 1:**
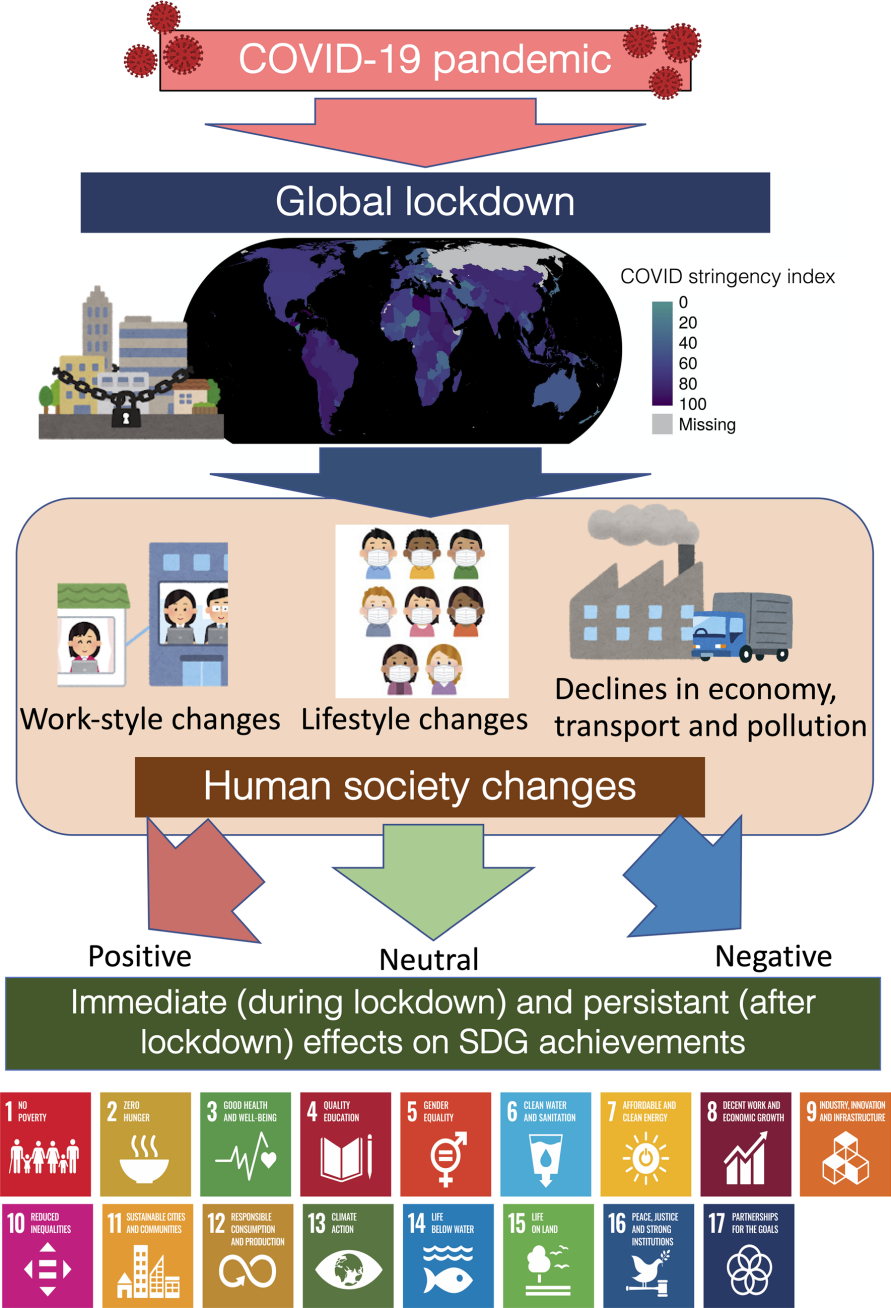
Conceptual illustration of the COVID-19 pandemic effect on SDG achievements through global lockdown consequences of societal changes. The COVID-19 GRSI is used as the lockdown degree index for each country on 15 June 2020. Illustrations are adopted from Irasutoya (https://www.irasutoya.com), except for SDG icons and the figure. SDG icons are adopted from https://www.globalgoals.org/resources

We first analysed how pandemic influenced the various quantitative factors, including the national lockdown policy, human movement, and nitrogen dioxide (NO_2_) emissions using an air pollution index based on database and satellite images from ‘before lockdown’ to ‘during lockdown’. Then, we presented how the global lockdown due to COVID-19 in early 2020 either enabled or inhibited the achievement of the SDGs either immediately or persistently (Fig. 1). Next, we analysed how the global lockdown influenced SDG achievements using our data and synthesised literature. We also assessed the immediate (during lockdown) and persistent (after lockdown) achievements for each SDG target using a simple assessment score employed in a previous study [16]. Finally, we have discussed the current and future influences of the global lockdown on society and the environment using the SDG scores and predict its persistent effects on SDG achievements.

## Materials and methods

### COVID-19 Government Response Stringency Index (GRSI)

We used data on the government responses to COVID-19, published by the Oxford COVID-19 Government Response Tracker (OxCGRT) [17]. OxCGRT collected COVID-19 GRSI daily from publicly available information for indicators, including ‘school closures’, ‘workplace closures’, ‘cancel public events’, ‘restrictions on gatherings’, ‘close public transport’, ‘public information campaigns’, ‘stay at home’, ‘restrictions on internal movement’, and ‘international travel controls’. The COVID-19 GRSI is a simple additive score of these nine indicators based on an ordinal scale from 0 to 100. The full details can be found at https://ourworldindata.org/grapher/covid-stringency-index.

We mapped the country-level COVID-19 GRSI data from 1 March 2020 to 1 June 2020 (Additional file 1: Fig. S1).

### Human migration

We used the data from the global mobility report published by Google (https://www.google.com/covid19/mobility/) to observe daily changes in human migration. This dataset describes changes in movements from the baseline, the median value of the five weeks from 3 January 2020 to 6 February 2020 (https://support.google.com/covid19-mobility/answer/9824897?hl=en&ref_topic=9822927). The mobility changes are classified into six categories: retail and recreation, grocery and pharmacy, parks, transit stations, workplaces, and residential. The data did not include any personally identifiable information, such as an individual’s location, contacts, or movement. Thus, the change values were built from aggregated and anonymised datasets of users who left their location history setting on for Google services, which is off by default. We used country-level mobility data for the six categories, collected from 15 February 2020 to 1 June 2020.

### NO_2_ emissions

We used satellite-based NO_2_ data observed by the TROPOspheric Monitoring Instrument (TROPOMI) onboard the Sentinel-5 Precursor launched by the European Space Agency with a spatial resolution of 0.01° as a proxy for air pollution data [18]. The data are available from July 2018 in the Google Earth Engine environment (https://earthengine.google.com), a planetary-scale cloud computing system for satellite imagery and geospatial datasets. To obtain NO_2_ data worldwide and visualise changes in NO_2_ emissions in response to the lockdown policies (e.g. from March 2020 to May 2020), the monthly median of the total vertical column of NO_2_ (the ratio of the NO_2_ slant column density and the total air mass factor) was calculated for every 0.01° grid in April 2019 and April 2020. Subsequently, we spatially aggregated NO_2_ emissions in each country and mapped the change rate ((NO_2__2020 – NO_2__2019)/NO_2__2019) for each country.

### Selecting SDG targets

The 17 SDGs are said to be ‘transforming our world’ and are a part of the United Nations’ 2030 Agenda for Sustainable Development [13]. The SDGs are associated with 169 targets, where each goal has 5 to 19 targets. We reviewed all 169 targets and selected those potentially influenced by the global lockdown due to the COVID-19 pandemic. The selection criteria were (1) the target could be influenced by human activities, including migration either directly or indirectly, and (2) we could evaluate the relationship between the accomplishment of targets and lockdown due to the COVID-19 pandemic. Also, we avoided targets that were conceptual, aimed to establish a social regulatory role, and aimed to get a social right. Consequently, we selected a total of 76 targets, of 17 which covered all goals.

### Scoring procedure to evaluate the effects of the lockdown

Lockdown policies have multiple levels [12]. We included all lockdown phenomena and policies that restricted society and behaviour in the scoring. We used a simple scoring system to evaluate the global lockdown effects on the achievement of each goal, and we calculated the conflict-synergy scores following the method used by Ibisch et al. [16].

The target score calculations were based on a simple index, where ‘negative’ indicated a negative influence, ‘positive’ indicated a positive influence, and ‘undecided’ indicated no influence or debating the pros and cons. On ‘undecided’, it has difficulty to discuss about that so we treat that as pending issue. The scores in each target could describe as:

− 1 (indicated by blue bar in Fig. 5): the lockdown negatively influence for the accomplishment on the target,
- 1 (indicated by red bar in Fig. 5): the lockdown positively influence for the accomplishment on the target,
- 0 (indicated by grey bar in Fig. 5): pending issue currently on the relationships with lockdown and the target.

In addition, we scored these indices according to two timescales: (1) immediate influence and (2) persistent influence. The scores of each SDG are based on a simple index comprising individual scores attributed to the corresponding targets.

We basically defined the score of each target that the literature review (including preprints and public reports) showed to be influenced by the lockdown. We could not conduct systematic literature survey because there were too many literatures on that topic (nature news, https://www.nature.com/articles/d41586-020-03564-y), and uncertainty regarding some effects remained [19]. Thus, we used descriptions to determine the target effects, and we did not use any specific cases. Some of the targets were clearly influenced by the lockdown. For example, Target 3 b, which aims to support vaccines and medicines for developing countries, can be directly influenced by COVID-19 issues including lockdown. In such cases, we attributed a score, even without consulting the literature. The literature survey was conducted during September 2020, and an additional survey to find the literature that could not be found the first time was conducted in both June and October 2021. This approach was found to be effective in all cases (Additional file 2: Table S1).

## Results

### Global lockdown and its consequences

**Fig. 2:**
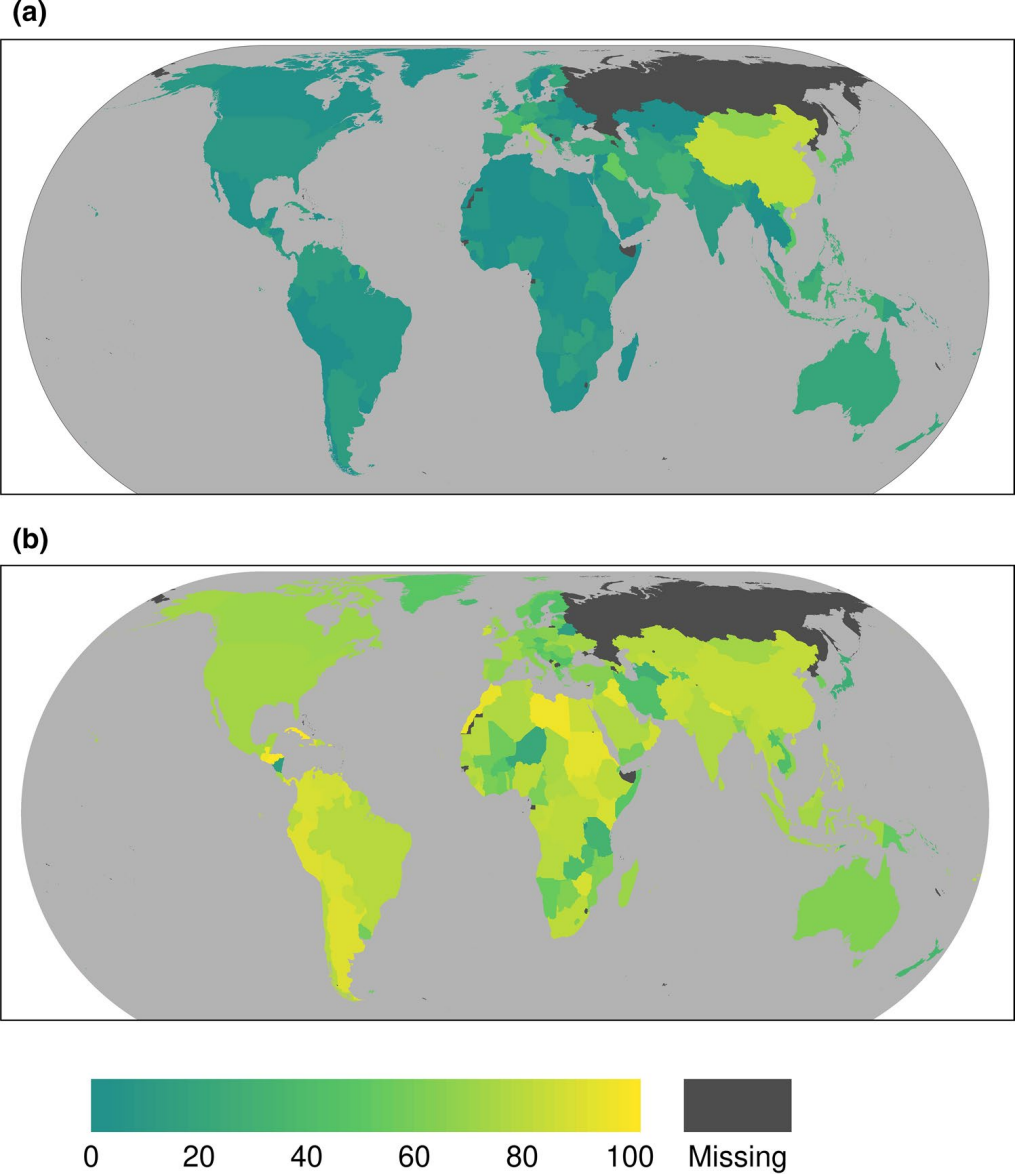
Country-level COVID-19 GRSI for **a** 1 March 2020 and **b** 1 June 2020. The index displays the degree of lockdown due to the COVID-19 pandemic

We analysed the lockdown policies, human movements, and NO_2_ emissions globally from February 2020 to June 2020. We exhibit the COVID-19 GRSI as a government policy response to COVID-19 [17] in Fig. 2. The COVID-19 GRSI scores were calculated daily based on citizen restriction policies. We found a higher GRSI score spread from China and its surrounding countries and increased from February 2020 to May 2020 (Fig. 2 and Additional file 1: Fig. S1). In Asian countries, the GRSI decreased in May 2020, while other countries maintained high GRSI values during this period.

**Fig. 3:**
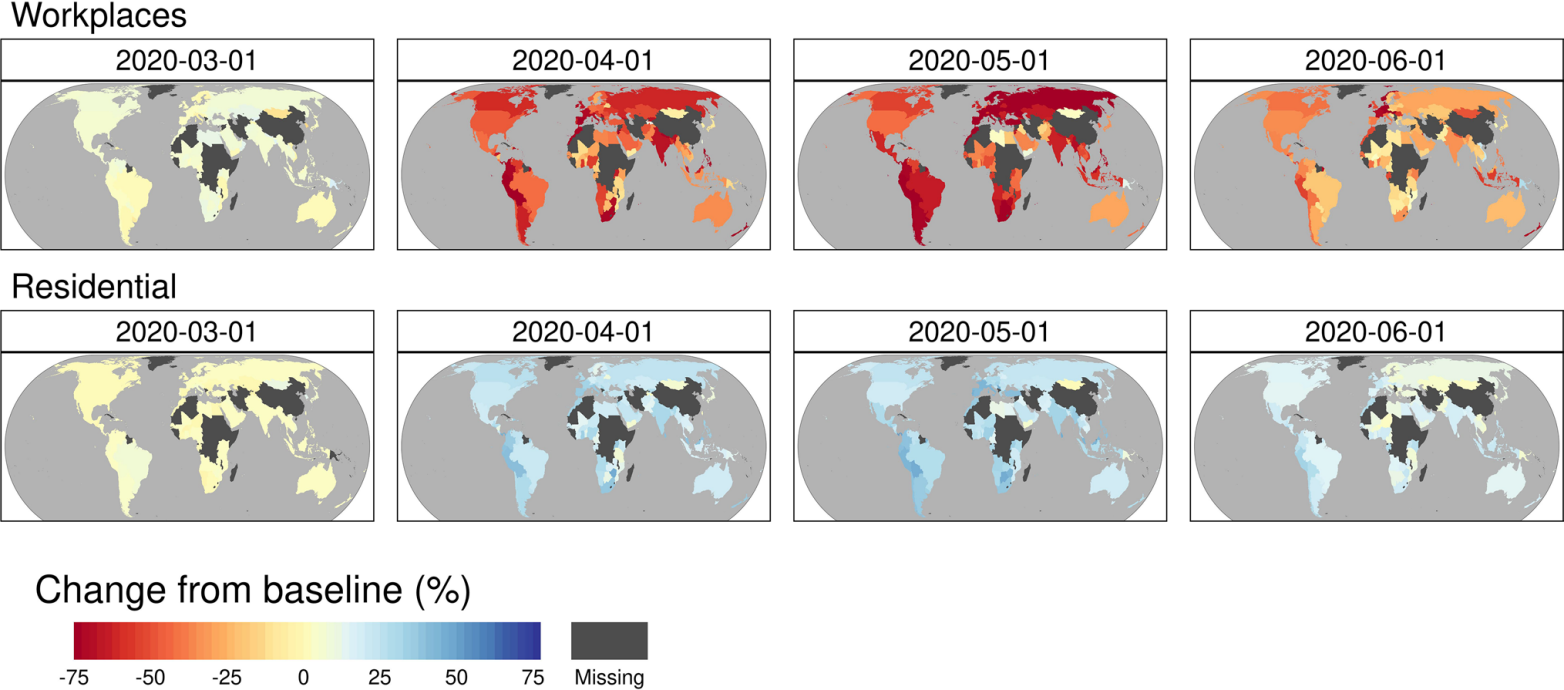
Mobility changes in the workplace and residential places from the baseline (the median value of the five weeks from 3 Jan 2020 to 6 February 2020) from 1 March 2020 to 1 June 2020

We illustrated global mobility changes to observe daily changes in human migration through Google services (Fig. 3 for the ‘workplaces’ and ‘residential’ categories). The ‘Workplace’ category was altered by − 80% in the measured countries, while the ‘residential’ category increased. The other mobility categories, including ‘retail and recreation’, ‘grocery and pharmacy’, ‘parks’, and ‘transit stations’, are displayed in Additional file 1: Fig. S2, and they also drastically decreased compared to the baseline after the global lockdown.

**Fig. 4:**
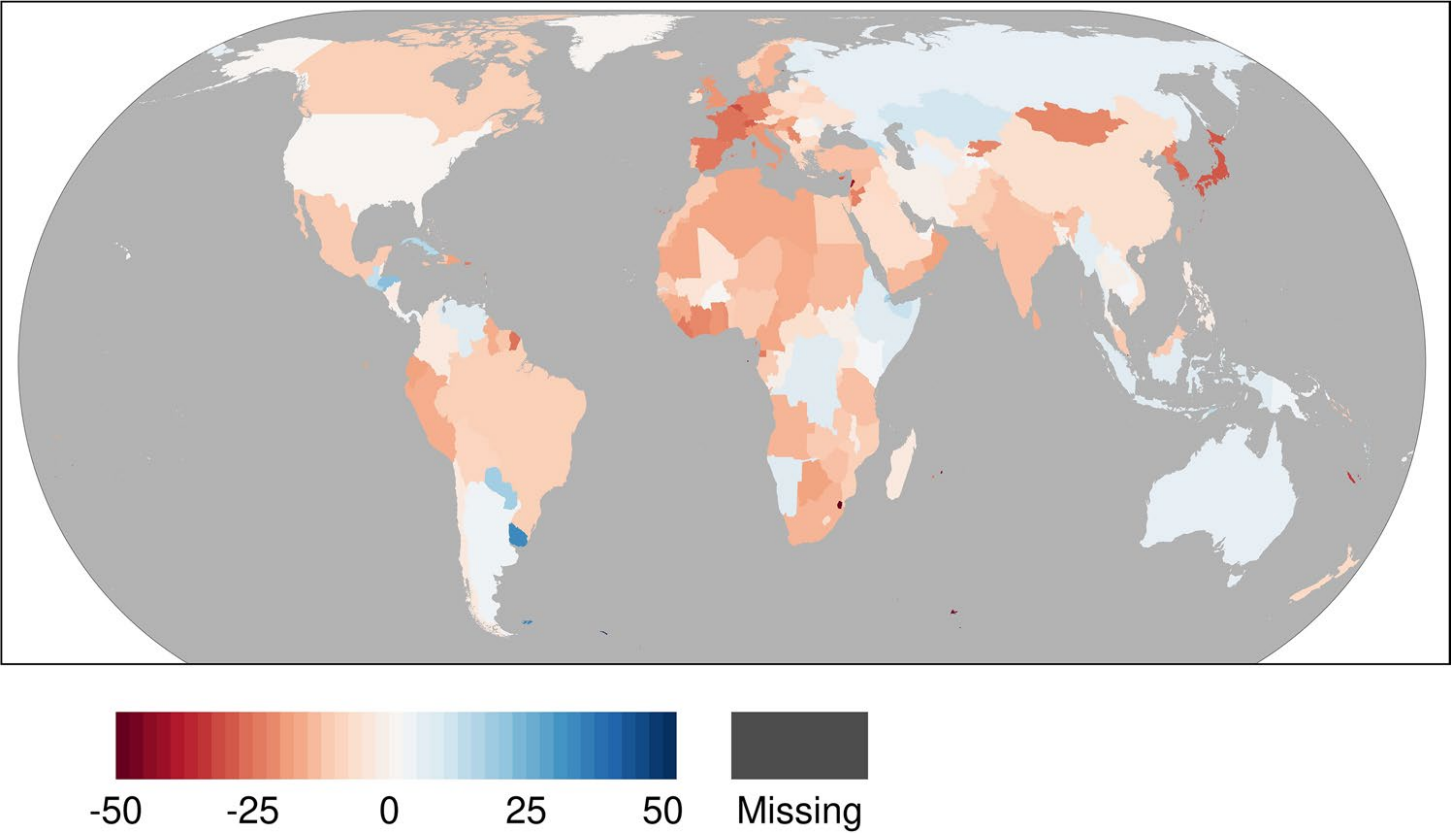
Change rate of country-level monthly median NO_2_ between April 2019 and April 2020

We calculated the change rate between the monthly median NO_2_ emission values for April 2019 and April 2020 for every country, and these are mapped in Fig. 4. We found that most countries in Europe, Asia, Africa, and North and South America exhibited negative change rates between 2019 and 2020. However, countries in Europe, Asia, and Africa exhibited higher change rates than those in other regions.

### SDG achievement scoring

**Fig. 5:**
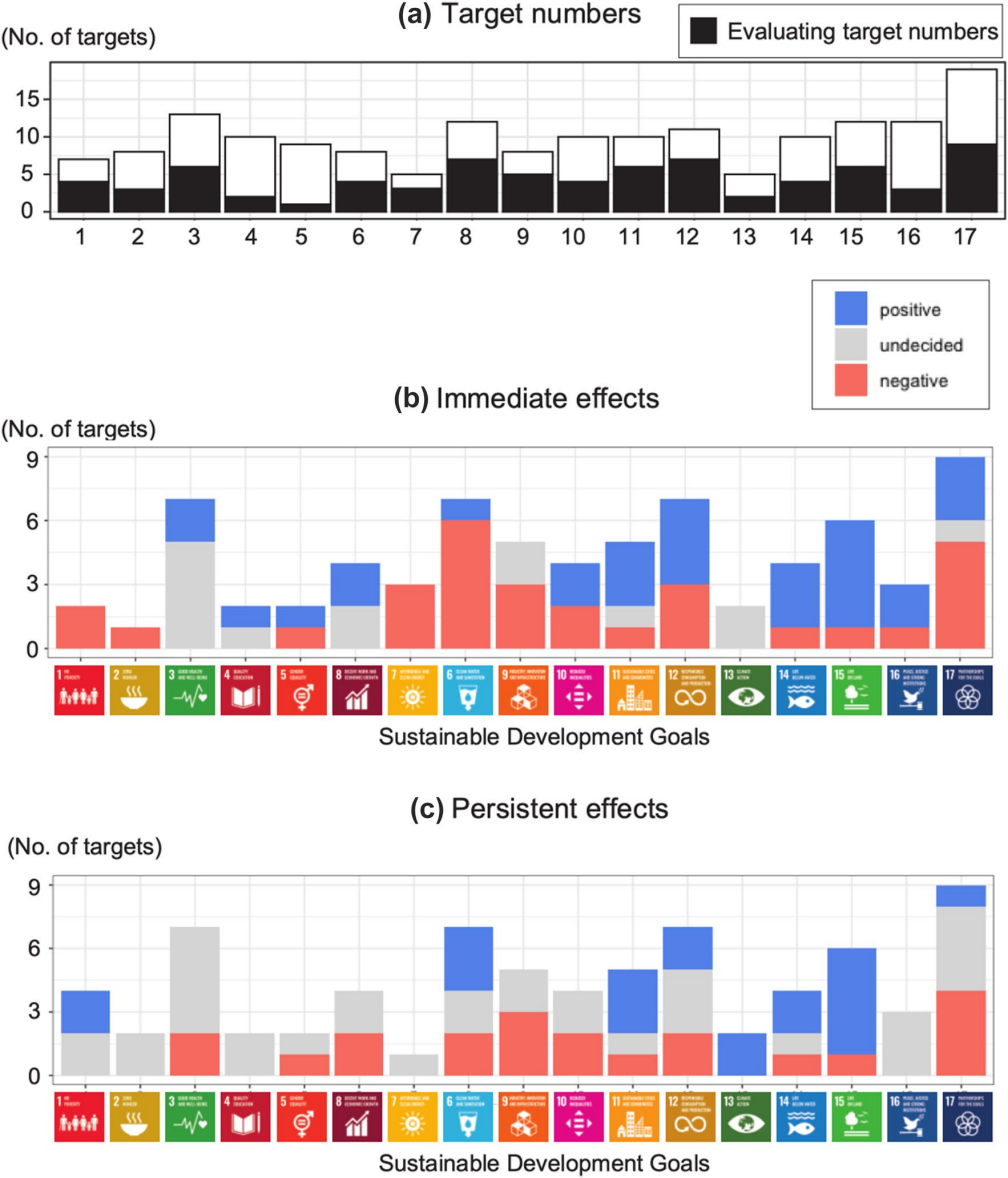
Scoring of global lockdown effects on SDG targets. **a** Evaluated targets for each goal. Scores of **b** immediate and **c** persistent effects of global lockdown on each goal

Using a simple scoring method, we summarised the positive, negative, and neutral effects of the COVID-19 lockdown on SDG achievements. We displayed the scoring of each goal (Table 1, Fig. 5) for the 73 targets (Fig. 5a). We found mixed negative and positive scores for SDG achievements when considering the immediate effects of the global lockdown (Fig. 5b). We observed many negative SDG target scores (Fig. 5b), such as those for SDGs 2 (zero hunger), 8 (decent work and economic growth), 9 (industry, innovation, and infrastructure), and 17 (partnerships). These mainly negatively affected SDGs concerned with food, economic, and industrial issues. Thus, these goals conflicted with the global lockdown. By contrast, SDGs 3 (good health and well-being), 6 (clean water and sanitation), 11 (sustainable cities and communities), 12 (responsible consumption and production), and 15 (life on land) exhibited more positive scores than the other Goals. These goals primarily concerned human health and environmental issues, including ecosystem conservation.

**Table 1:** Scoring of global lockdown effects on SDG targets (the scores of immediate and persistent effects of global lockdown on each goal)

Goals	Immediate effect			Persistent effect			Evaluated
	Negative	Neutral	Positive	Negative	Neutral	Positive	
1	2	2	0	2	0	2	4
2	1	0	0	0	0	2	2
3	0	5	2	0	2	5	7
4	0	1	1	0	0	2	2
5	1	0	1	0	1	1	2
6	0	2	2	0	2	2	4
7	3	0	0	0	0	1	1
8	6	0	1	3	2	2	7
9	3	2	0	0	3	2	5
10	2	0	2	0	2	2	4
11	1	1	3	3	1	1	5
12	3	0	4	2	2	3	7
13	0	2	0	2	0	0	2
14	1	0	3	2	1	1	4
15	1	0	5	5	1	0	6
16	1	0	2	0	0	3	3
17	5	1	3	1	4	4	9

Evaluated means number of the evaluated targets in each Goal

When considering the persistent effects of the COVID-19 pandemic on SDG achievements, Goal 6 exhibited the same score as that of the immediate effect (Fig. 5b). The other SDGs exhibited different achievements compared with their immediate responses (Fig. 5b and c). In particular, the scores of SDGs 11, 13 (Climate action), and 15 mostly shifted from positive to negative, which may be due to the recovery of human activities, including the economy. By contrast, SDGs 9 and 10 (reduced inequalities), 16 (peace, justice, and strong institutions), and 17 predominantly changed from negative to positive, which may be due to improved governance. The detailed responses for each target were displayed in Additional file 2: Table S1. Several SDG targets showed ambivalent (instead of synergistic) effects of the global lockdown.

## Discussion

We evaluated the effects of the global lockdown on society and the environment. We found drastic changes in government policies in response to COVID-19, such as increasing expenditures, reducing human movement, reducing human mobility/working style, and consequently reducing air pollution, as evidenced by NO_2_ data. The reduction in air pollution may result from the reduction in economic and transportation activities [9, 19–21]. For example, the absence of motor vehicle traffic and suspended manufacturing during the COVID-19 pandemic in China led to a ~ 90% reduction in NO_2_ emissions countrywide [22]. The global economy drastically slowed due to the COVID-19 pandemic [23]. As the changes in NO_2_ emissions are consistent with those in other emissions such as carbon dioxide (CO_2_), ozone (O_3_), and fine particulate matter (PM_2.5_) [20, 24, 25], the NO_2_ emission level can be regarded as an air pollution indicator. Thus, we found the degree of reduction in air pollution response to the lockdown as a proxy through the NO_2_ observation. Most countries in the world reduced air pollution through their lockdown policies (Fig. 2b).

These changes began in early March 2020 after COVID-19 spread globally. Such global changes in society due to a global lockdown have not been observed previously due to limited observation techniques. In this study, we originally examined the global lockdown consequences for human movement and environmental impacts using current technologies, such as human location big data via smartphone and satellite images. Global consequences of the lockdown have been observed for other phenomena, such as CO_2_ emissions [9], air PM_2.5_ concentration [26], human mobility via ‘Disease Prevention Maps’ by Facebook users [27], and environmental noise [28]. Against these previous works, we here provided new consequences of the global lockdown using new scoring and data of NO_2_ emissions, with results similar to those of previous reports.

The COVID-19-induced lockdown may negatively affect the achievement of the SDGs concerning food, the economy, and infrastructure (e.g. Goals 3 and 9). The lockdown is expected to substantially influence the food supply chain [29], infrastructure, and the economy [23] due to restricted human movement, food production, and economic activities. The global lockdown may accelerate the achievements of certain SDGs, especially those focused on improving human health and conserving ecosystems. Especially for food security, some studies have assessed the impact of the COVID-19 pandemic on food security and resulting health effects [30–32]. Galanakis [33] surmised that the COVID-19 pandemic had created a new era for food-supply chains. We will have to face many significant challenges, e.g., ensuring food safety and security, reducing losses and food wastage, as well as identifying alternative, safe protein sources that meet the nutritional expectations of consumers.

The global lockdown may reduce global climate change impact due to the relative decline in air pollution (Goal 13) [9, 26], conserve sustainable cities [11], and protect life on land [15] through a lower human impact on ecosystems [5]. Consequently, human health may improve, excluding those who contract COVID-19 (Additional file 2: Table S1). These improvements are primarily due to restricted human movements and economic activities, which reduce air pollution. Certain scholars expected reduced human mobility and activity during the global lockdown to significantly impact ecosystems because reducing the human impact on the environment allows ecosystems to recover and conserves species [4, 34].

Regarding persistent effects on SDG achievements up to 2030, the SDG scores changed drastically from the immediate ones. The achievements of climate change and ecosystem protection, such as Goals 11, 13, and 15, predominantly shifted from positive to negative, while those of economic issues, such as Goals 9, 10, and 16, primarily exhibited a negative to positive trend. This occurred primarily because economic recovery that goes against ecosystem protection can be expected to occur after the global lockdown. Forster et al. [35] simulated the increase in global temperature after the economic recovery up to 2030. Therefore, global economic recovery could substantially affect the persistent achievements of the SDGs concerning ecosystem protection and climate change mitigation. Furthermore, there were conflicting effects among goals protecting biodiversity and those promoting economic development [16, 36]. Therefore, a comprehensive debate is necessary to consider the achievements of SDGs concerning economic recovery and ecosystem management after global lockdown.

There is a growing body of scientific information on how to achieve SDGs [16, 37–39], and the impacts of the global lockdown have been well evaluated using the current global policy framework. Moreover, major global lockdown policies and the subsequent economic recovery, such as the cohesion policy of developed countries, may not consider the future SDG achievements for 2030. Therefore, policies concerning SDGs should be considered while factoring in the global lockdown and subsequent economic recovery.

This study design and the perspectives have certain limitations. Our simple score analysis for SDG achievements represents findings in the literature on how the global lockdown affects the SDGs. Although we carefully considered the scores, certain scores may have been overlooked or underestimated. In addition, we should assume that new evidence of the global lockdown effects on SDGs will be published in the future. Considering these limitations, the scoring estimates have various uncertainties. Therefore, we recommend studying the reality of SDG achievement in the future by directly measuring the SDGs, human movement, and air pollution. We scored the SDG achievement at the global scale by limiting the data; however, developing countries are more severely influenced by the global lockdown due to their limited governmental budgets [40]. Therefore, we encourage the assessment of SDG achievements at the country or regional level.

Recently, with increasing levels of COVID-19 vaccination, the lockdown has been reduced in parts of the world. The changing lockdown situation could improve or reduce the SDG achievements at the country scale as well as the global scale. Moreover, use of resources for the COVID-19 pandemic, e.g., lockdown, is likely to hinder reactions to concurrent threats (e.g., heat waves, wildfires, drought, and extreme weather) as under-resourced systems and emergency responses become stretched and disrupted [41], radically transforming the current state of global development [42]. Such threats increase the potential for geopolitical unrest, and the cost of dealing with these stressors could divert funding from the existing SDG targets [6].

In conclusion, we have highlighted the changes in society, the environment, and SDG achievements due to the immediate and persistent effects of the global lockdown caused by the COVID-19 pandemic. Although our simple score analysis scored the SDG achievement by limiting the data and containing any uncertainly, global lockdown significantly impacted society and the environment. Also, our study is a first step to describe the time-series observation by satellite and the prediction for SDGs achievement, but time-series analysis for the air pollution [43] and the scenario analysis for prediction [44] would be helpful to further analysis and consequently policy making under the COVID-19 lockdown. Moreover, it greatly impacted the immediate achievements of the most SDGs, with mainly negative and positive effects on economic and environmental issues, respectively. In addition, we found there were persistent effects on achievements for most of the goals. We are at a critical turning point for the future of human society and the Earth, and the SDG achievement analysis provides powerful evidence for this from the SDG perspective [39]. In addition, the SDGs represent a leap forward compared to the Millennium Development Goals [45]. Humanity is currently facing the COVID-19 pandemic; however, to achieve a sustainable society shared principles and legislation among nations must be developed. The political choices made during and after the COVID-19 pandemic could potentially assist the development of a sustainable society by 2030 according to he SDG achievement as we partly predicted.

## Supplementary Information

Below is the link to the electronic supplementary material.

Supplementary file1 (DOCX 2939 KB)

Supplementary file2 (DOCX 89 KB)

## Data Availability

We used the data from the Oxford COVID-19 Government Response Tracker (OxCGRT), the global mobility report openly published by Google (https://www.google.com/covid19/mobility/), and Google Earth Engine environment (https://earthengine.google.com).
