# Supplementary material for: Assessing the potential repercussions of the COVID-19 pandemic on global SDG attainment
Source: Discov Sustain. 2022 Jan 18;3(1):2. doi: 10.1007/s43621-021-00067-2 (PMC8765102; doi:10.1007/s43621-021-00067-2)
Supplement: Supplementary file 1 — Supplementary file1 (DOCX 2939 KB) [file 43621_2021_67_MOESM1_ESM.docx]

Supplementary Materials

**Global lockdown potential impact on achieving Sustainable Development Goals**

Hideyuki Doi, Takeshi Osawa, and Narumasa Tsutsumida


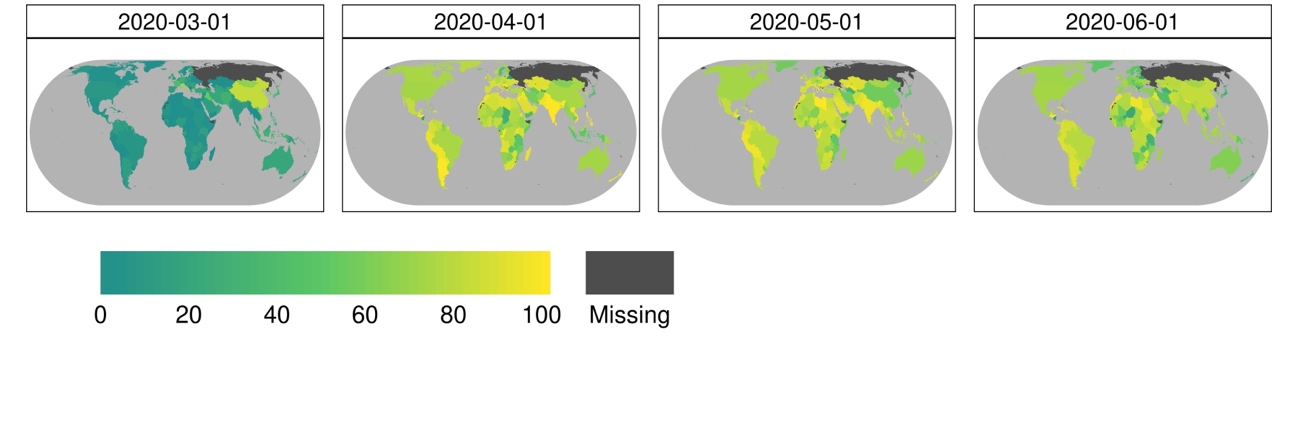


Figure S1 Country-level COVID-19 GRSI from 1 March 2020 to 1 June 2020. The index illustrated the degree of lockdown for the COVID-19 pandemic.


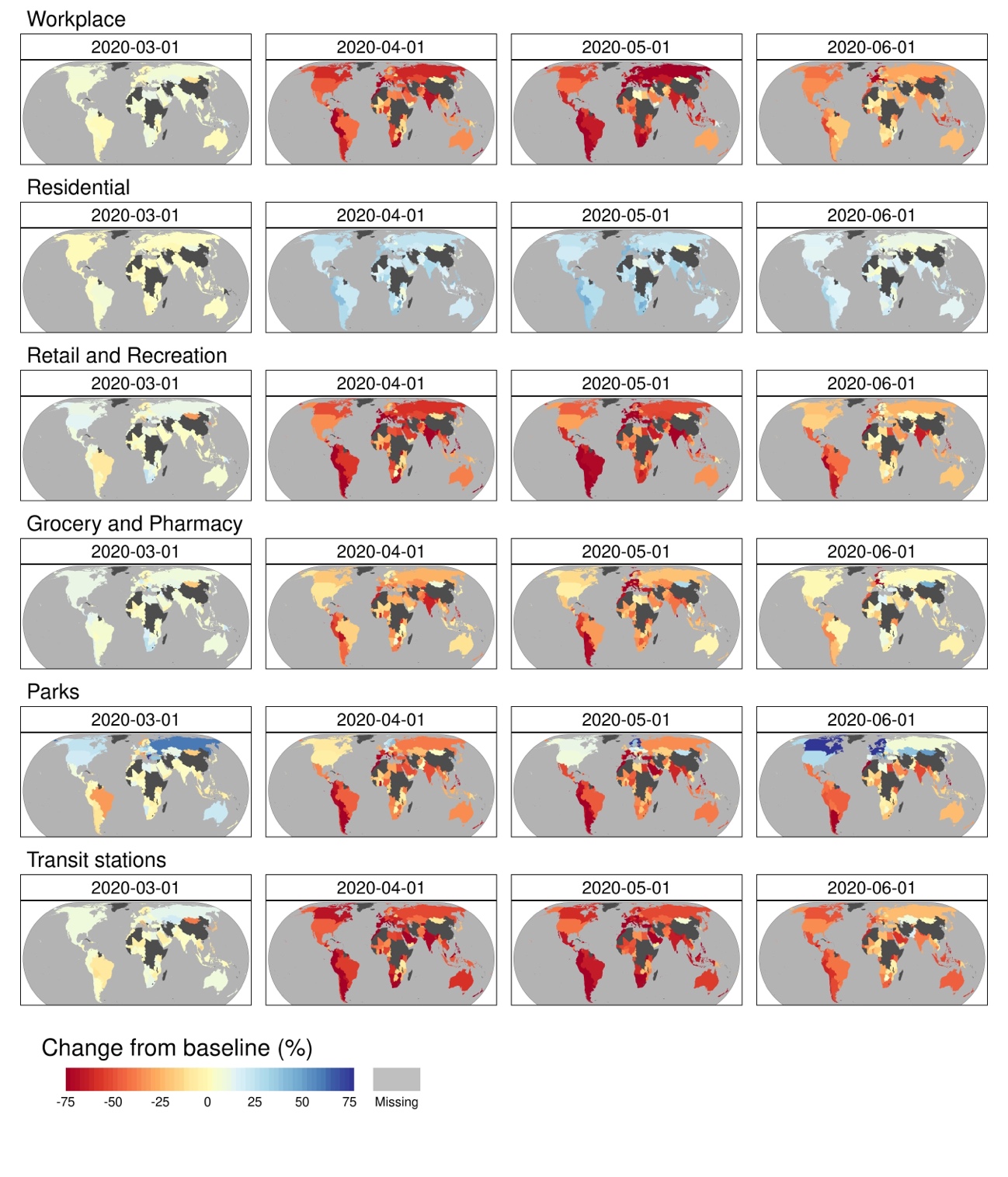


Figure S2. Mobility changes in the workplace, residential areas, retail and recreation, grocery and pharmacy, parks, and transit stations from the baseline (the median value is from the five weeks from 3 January 2020 to 6 February 2020), from 1 March 2020 to 1 June 2020.
