# Supplementary material for: Assessing the potential repercussions of the COVID-19 pandemic on global SDG attainment
Source: Discov Sustain. 2022 Jan 18;3(1):2. doi: 10.1007/s43621-021-00067-2 (PMC8765102; doi:10.1007/s43621-021-00067-2)
Supplement: Supplementary file 2 — Supplementary file2 (DOCX 89 KB) [file 43621_2021_67_MOESM2_ESM.docx]

**Table S1. SDG target scoring results**

| **Immediate and persistent effect scores for each SDG** | **Target evaluations** |
| --- | --- |
| **Goal 1. End poverty in all its forms everywhere**   \|  \| **Positive** \| **Undecided** \| **Negative** \| \| --- \| --- \| --- \| --- \| \| **Immediate effect** \| **0** \| **0** \| **2** \| \| **Persistent effect** \| **2** \| **0** \| **2** \| | Immediate effect:  (1.1 and 1.2) (negative)  Lockdown reduces economic activities ^1–4^, which negatively influences poverty.  Persistent effect:  (1.1 and 1.2) (negative)  Decreasing economic activities negatively influence poverty in the long term ^1–4^.  (1.3) (positive)  Governments should take aggressive countermeasures against the increasing vulnerable person caused by social protection systems ^3^; thus, there may be a positive influence as a persistent ^4^.  (1.5) (positive)  The COVID-19 pandemic, sagging economy caused by lockdown expand extreme poverty (World Bank: <http://www.hlrn.org/activitydetails.php?id=pnFpZA==#.YXkMihxUtaQ>; accessed at 21 Dec 2021). That exposed many vulnerabilities for poor people; thus, there may be a positive influence on poverty in the long term. Actually, World Bank called for collective action to reduce poverty. |
| **Goal 2. End hunger, achieve food security and improved nutrition, and promote sustainable agriculture**   \|  \| **Positive** \| **Undecided** \| **Negative** \| \| --- \| --- \| --- \| --- \| \| **Immediate effect** \| **0** \| **0** \| **1** \| \| **Persistent effect** \| **2** \| **0** \| **0** \| | Immediate effect:  (2.1 and 2.c) (negative)  Reducing transportation caused food distribution system issues, negatively influencing hunger ^5,6^.  Persistent effect:  (2.1 and 2.c) (positive)  After lockdown, food security should rapidly recover ^5,6^. |
| **Goal 3. Ensure healthy lives and promote wellbeing for all at all ages**   \|  \| **Positive** \| **Undecided** \| **Negative** \| \| --- \| --- \| --- \| --- \| \| **Immediate effect** \| **2** \| **5** \| **0** \| \| **Persistent effect** \| **5** \| **2** \| **0** \| | Immediate effect:  (3.6) (positive)  Lockdown causes a drastic reduction in transportation ^7^.  (3.9) (positive)  Lockdown mitigates air pollution primarily caused by transportation ^7^.  Persistent effect:  (3.3 and 3.8) (positive)  Lockdown caused active discussion about improving medical resources re-distribution ^8^.  (3.b) (positive)  Lockdown motivates research for developing vaccine and distribution.  (3.c) (positive)  Lockdown enhances concern for public health worldwide.  (3.d) (positive)  People discovered the importance of warning systems for a pandemic ^9^. |
| **Goal 4. Ensure inclusive and equitable quality education and promote lifelong learning opportunities for all**   \|  \| **Positive** \| **Undecided** \| **Negative** \| \| --- \| --- \| --- \| --- \| \| **Immediate effect** \| **1** \| **1** \| **0** \| \| **Persistent effect** \| **2** \| **0** \| **0** \| | Immediate effect:  (4.1) (positive)  Lockdown expands the on-line education systems ^10^.  Persistent effect:  (4.1) (positive)  The home-study system is an important legacy of the lockdown ^10^.  (4.6) (positive)  On-line education systems can provide opportunities to bring education to children with difficulties in accessing the education system ^10^. |
| **Goal 5. Achieve gender equality and empower all women and girls**   \|  \| **Positive** \| **Undecided** \| **Negative** \| \| --- \| --- \| --- \| --- \| \| **Immediate effect** \| **1** \| **0** \| **1** \| \| **Persistent effect** \| **1** \| **0** \| **0** \| | Immediate effect:  (5.1) (negative)  Females are more likely to be affected by the COVID-19 lockdown because of their typical occupations ^11^.  (5.4) (positive)  Lockdown increased the amount of time spent at home, which altered how household work is shared within a family independent of gender.  Persistent effect:  (5.4) (positive)  Staying home for a longer period can result in the continued sharing of housework within a family independent of gender. Mitsubishi UFJ Research and Consulting Co. reported about 50 % of Japanese teleworker were increasing the time of housework (https://www.murc.jp/wp-content/uploads/2021/03/seiken_210301.pdf, in Japanese). |
| **Goal 6. Ensure availability and sustainable management of water and sanitation for all**   \|  \| **Positive** \| **Undecided** \| **Negative** \| \| --- \| --- \| --- \| --- \| \| **Immediate effect** \| **2** \| **2** \| **0** \| \| **Persistent effect** \| **2** \| **2** \| **0** \| | Immediate effect:  (6.3) (positive)  Lockdown mitigates water pollution primarily caused by human activities ^12^.  (6.6) (positive)  Lockdown mitigates environmental pressure by decreasing human activities ^12^.  Persistent effect:  (6.1 and 6.2) (positive)  The lockdown and COVID-19 result in a focus on water treatment for public health ^13^. |
| **Goal 7. Ensure access to affordable, reliable, sustainable, and modern energy for all**   \|  \| **Positive** \| **Undecided** \| **Negative** \| \| --- \| --- \| --- \| --- \| \| **Immediate effect** \| **0** \| **0** \| **3** \| \| **Persistent effect** \| **1** \| **0** \| **0** \| | Immediate effect:  (7.1, 7.2 and 7.a) (negative)  Lockdown and COVID-19 itself collapsed fossil fuel prices, especially renewable energy were losing their competitiveness^14,15^.  Persistent effect:  (7.a) (positive)  Hospitality businesses investment usually rewards slowly but constantly and that conducted during a pandemic could be effective ^16^. |
| **Goal 8. Promote sustained, inclusive, and sustainable economic growth, full and productive employment, and decent work for all**   \|  \| **Positive** \| **Undecided** \| **Negative** \| \| --- \| --- \| --- \| --- \| \| **Immediate effect** \| **1** \| **0** \| **6** \| \| **Persistent effect** \| **2** \| **2** \| **3** \| | Immediate effect:  (8.1, 8.2, 8.3, 8.8, and 8.9) (negative)  Economic damage caused by lockdown reduces economic developments including technology and policy improvements ^1^.  (8.4) (positive)  A difficult economic situation reduces environmental pressure and requires the promotion of streamlining ^7,12^.  Persistent effect:  (8.1 and 8.2) (negative)  The economic damages due to the lockdown cannot recover in the short term ^1^.  (8.3 and 8.9) (positive)  Lockdown may enhance economic decentralization ^1^.  (8.4) (negative)  After the lockdown, society can invest in economic recovery without considering environmental pressures ^1^. |
| **Goal 9. Build resilient infrastructure, promote inclusive and sustainable industrialization, and foster innovation**   \|  \| **Positive** \| **Undecided** \| **Negative** \| \| --- \| --- \| --- \| --- \| \| **Immediate effect** \| **0** \| **2** \| **3** \| \| **Persistent effect** \| **2** \| **3** \| **0** \| | Immediate effect:  (9.1, 9.2, and 9.3) (negative)  Economic damages due to lockdown and COVID-19 negatively influence the development of infrastructure, industry, and money and banking activities ^1^.  Persistent effect:  (9.4 and 9.5) (positive)  After the lockdown and pandemic, society can invest in both sustainable infrastructure and industrial developments. |
| **Goal 10. Reduce inequality within and among countries**   \|  \| **Positive** \| **Undecided** \| **Negative** \| \| --- \| --- \| --- \| --- \| \| **Immediate effect** \| **2** \| **0** \| **2** \| \| **Persistent effect** \| **2** \| **2** \| **0** \| | Immediate effect:  (10.1) (negative)  Economic damages from lockdown and COVID-19 negatively influence income and employment ^1^.  (10.4 and 10.5) (positive)  Certain countries have enacted policies that support the economic and social security of all citizens such as benefit money and evaluated their effectiveness ^17^.  (10.7) (negative)  Emigrant, immigrant, and overseas educations are expected to be inhibited by the lockdown.  Persistent effect:  (10.4 and 10.5) (positive)  Enacting policies to improve the economic and social security of all citizens can continue ^17^. |
| **Goal 11. Make cities and human settlements inclusive, safe, resilient, and sustainable**   \|  \| **Positive** \| **Undecided** \| **Negative** \| \| --- \| --- \| --- \| --- \| \| **Immediate effect** \| **3** \| **1** \| **1** \| \| **Persistent effect** \| **1** \| **1** \| **3** \| | Immediate effect:  (11.2) (negative)  The lockdown affects the public transportation system independent of social status ^18,19^.  (11.4) (positive)  Lockdown reduces tourism activities including visits to world heritage sites ^18–20^.  (11.6) (positive)  Lockdown reduces environmental pressures, especially in urban regions ^12,21,22^.  (11.7) (positive)  Lockdown enhances access to green spaces ^23,24^.  Persistent effect:  (11.4) (negative)  After the lockdown, tourism activities can recover quickly ^20^.  (11.5) (negative)  After lockdown, governments may focus on policy measures concerning pandemics, not natural disasters.  (11.6) (negative)  After the lockdown, economic activities can increase, which raises environmental pressures ^25^.  (11.7) (positive)  The lockdown may result in enhanced access to green spaces even after it has concluded ^23,24^. |
| **Goal 12. Ensure sustainable consumption and production patterns**   \|  \| **Positive** \| **Undecided** \| **Negative** \| \| --- \| --- \| --- \| --- \| \| **Immediate effect** \| **4** \| **0** \| **3** \| \| **Persistent effect** \| **3** \| **2** \| **2** \| | Immediate effect:  (12.2) (negative)  Lockdown reduces the number of international supply chains that are in turmoil, improving resource management ^26^.  (12.3) (positive)  Lockdown puts many restaurants out of business, thus, food waste should be decreased.  (12.4 and 12.5) (positive)  The lockdown caused an industrial shutdown, which temporarily reduced emissions ^27,28^.  (12.6) (negative)  The lockdown caused a temporal industrial shutdown for international companies regardless of size ^27,28^.  (12.7) (negative)  The lockdown inhibits the enforcement of domestic policy.  (12.8) (positive)  The lockdown resulted in the replacement of face-to-face activities with virtual platforms for a wide range of social activities ^26^.  Persistent effect:  (12.4 and 12.6) (negative)  An industrial shutdown by lockdown is temporal; thus, these activities should be active in the long term ^27,28^.  (12.6) (positive)  The shutdown recovery process can enhance international collaboration.  (12.7) (positive)  The lockdown can create an international trend of aspiring to consume sustainably ^26^.  (12.8) (positive)  Lockdown can enhance the change from face-to-face activities to virtual platforms for a wide range of social activities ^26^. |
| **Goal 13. Take urgent action to combat climate change and its impacts**   \|  \| **Positive** \| **Undecided** \| **Negative** \| \| --- \| --- \| --- \| --- \| \| **Immediate effect** \| **0** \| **2** \| **0** \| \| **Persistent effect** \| **0** \| **0** \| **2** \| | Immediate effect:  There is no evidence of a change in SDG achievement.  Persistent effect:  (13.1) (negative)  After lockdown, society will likely focus on economic recovery, not natural disasters.  (13.2) (negative)  The recovery process of industrial activities may not focus on climate change strategies ^3,20^. |
| **Goal 14. Conserve and sustainably use the oceans, seas, and marine resources for sustainable development**   \|  \| **Positive** \| **Undecided** \| **Negative** \| \| --- \| --- \| --- \| --- \| \| **Immediate effect** \| **3** \| **1** \| **1** \| \| **Persistent effect** \| **1** \| **1** \| **2** \| | Immediate effect:  (14.1) (negative)  The lockdown caused an increase in the amount of medical waste in the ocean ^12^.  (14.2, 14.3) (positive)  The lockdown caused a shutdown of fisheries, decreasing marine traffic and reducing the pressure on marine ecosystems ^29,30^.  (14.4) (positive)  The lockdown caused fisheries to shut down ^29^.  Persistent effect:  (14.1) (negative)  The increasing amount of medical waste in the ocean may be prolonged until the end of the pandemic ^12^.  (14.2) (positive)  Fishery recovering policy has not been fully discussed, implying that they may not recover quickly ^29^.  (14.3) (negative)  Marine traffic can recover after the global lockdown ^30^. |
| **Goal 15. Protect, restore, and promote sustainable use of terrestrial ecosystems, sustainably manage forests, combat desertification, and halt and reverse land degradation and biodiversity loss**   \|  \| **Positive** \| **Undecided** \| **Negative** \| \| --- \| --- \| --- \| --- \| \| **Immediate effect** \| **5** \| **0** \| **1** \| \| **Persistent effect** \| **0** \| **1** \| **5** \| | Immediate effect:  (15.1, 15.2, 15.4, and 15.5) (positive)  The lockdown resulted in traffic control, industry shutdowns, and movement restrictions, which strongly reduced environmental pressures ^7,12,18,19,31,32^.  (15.7) (negative)  The lockdown interrupted conservation activities ^32,33^.  (15.8) (positive)  The lockdown caused traffic control and movement restrictions that strongly reduced invasive species introductions ^7,12,18,19,31,32–30^.  Persistent effect:  (15.1, 15.2, 15.4, and 15.5) (negative)  Lockdown effects for human activities such as traffic control, industry shutdowns, and movement restrictions are temporal; thus, these activities can be recovered ^7,12,18,19,31^.  (15.8) (negative)  The recovery process of traffic control and movement restrictions can be expected to introduce non-native species to new habitats. |
| **Goal 16. Promote peaceful and inclusive societies for sustainable development, provide access to justice for all, and build effective, accountable, and inclusive institutions at all levels**   \|  \| **Positive** \| **Undecided** \| **Negative** \| \| --- \| --- \| --- \| --- \| \| **Immediate effect** \| **2** \| **0** \| **1** \| \| **Persistent effect** \| **3** \| **0** \| **0** \| | Immediate effect:  (16.6) (positive)  National policy concerning public sector homologies can be required during the lockdown.  (16.7) (negative)  National policy concerning public sector homologies can be negatively affected by the lockdown.  (16.10) (positive)  The lockdown increased the use of online platforms, which can improve information accessibility ^26^.  Persistent effect:  (16.6 and 16.7) (positive)  National policy concerning public sector homologies can mitigate the damage caused by the lockdown; thus, enhanced governance is a key issue for the future.  (16.10) (positive)  The lockdown increases the use of online platforms that can improve information accessibility ^26^. |
| **Goal 17. Strengthen the means of implementation and revitalize the global partnership for sustainable development**   \|  \| **Positive** \| **Undecided** \| **Negative** \| \| --- \| --- \| --- \| --- \| \| **Immediate effect** \| **3** \| **1** \| **5** \| \| **Persistent effect** \| **4** \| **4** \| **1** \| | Immediate effect:  (17.1) (negative)  The lockdown caused severe financial damages to countries.  (17.2) (negative)  Economic damages due to the lockdown ^1–4^ can directly influence financial support including Official Development Assistances (ODAs). Actually, aid data already showed some donors falls in aid over 40 %.  (17.6) (positive)  International collaboration for COVID-19 is expected to increase during the lockdown via on-line communication. US and China strengthen their research relationship even each has strong lockdown (Fry et al. 2020; Consolidation in a crisis: Patterns of international collaboration in early COVID-19 research, ).  (17.8) (positive)  The lockdown enhances the use of online platforms ^26^ including that in educational systems ^10^.  (17.11, 17.12, and 17.13) (negative)  The lockdown caused international trade to shrink ^1^.  (17.18) (positive)  Political actions including the lockdown decision required key statistics ^34^.  Persistent effect:  (17.2) (negative)  Economic damages due to the lockdown ^1–4^ can directly influence financial support including ODAs.  (17.6) (positive)  International collaboration is projected to increase even after the lockdown via on-line communication. International Chamber of Commerce (ICC) calls for international cross-border scientific collaboration.  (17.8) (positive)  The lockdown can enhance the use of online platforms ^26^ including that in educational systems ^10^.  (17.14) (positive)  Although there are a variety of policies in various countries ^1,35^, all countries are focusing on economic problems. One clear example is ODA, that influence not only developed country also developing country.  (17.18) (positive)  Political actions including the lockdown decision required key statistics ^34^. |

References

1. McKibbin, W. J. & Fernando, R. The Global Macroeconomic Impacts of COVID-19: Seven Scenarios. *SSRN Electron. J.* (2020) doi:10.2139/ssrn.3547729.

2. Sumner, A., Hoy, C. & Ortiz-juarez, E. Wider Working Paper 2020 / 43 Estimates of the impact of Covid-19 on global poverty. *WIDER Work. Pap. 2020/43* **43**, 1–14 (2020).

3. Nicola, M. *et al.* The socio-economic implications of the coronavirus pandemic (COVID-19): A review. *Int. J. Surg.* **78**, 185–193 (2020).

4. Adam, C., Henstridge, M. & Lee, S. After the lockdown: macroeconomic adjustment to the COVID-19 pandemic in sub-Saharan Africa. *Oxford Rev. Econ. Policy* **36**, S338–S358 (2020).

5. Loopstra, R. Vulnerability to food insecurity since the COVID-19 lockdown Preliminary report. *Food Found.* (2020).

6. The Lancet. India under COVID-19 lockdown. *Lancet* **395**, 1315 (2020).

7. Muhammad, S., Long, X. & Salman, M. COVID-19 pandemic and environmental pollution: A blessing in disguise? *Sci. Total Environ.* **728**, 138820 (2020).

8. Emanuel, E. J. *et al.* Fair allocation of scarce medical resources in the time of covid-19. *N. Engl. J. Med.* **382**, 2049–2055 (2020).

9. Ho, C. S., Chee, C. Y. & Ho, R. C. Mental Health Strategies to Combat the Psychological Impact of COVID-19 Beyond Paranoia and Panic. *Ann. Acad. Med. Singapore* **49**, 1–3 (2020).

10. Wang, G., Zhang, Y., Zhao, J., Zhang, J. & Jiang, F. Mitigate the effects of home confinement on children during the COVID-19 outbreak. *Lancet* **395**, 945–947 (2020).

11. Wenham, C., Smith, J. & Morgan, R. COVID-19: the gendered impacts of the outbreak. *Lancet* **395**, 846–848 (2020).

12. Saadat, S., Rawtani, D. & Hussain, C. M. Environmental perspective of COVID-19. *Sci. Total Environ.* **728**, 138870 (2020).

13. WHO. Water , sanitation , hygiene and waste management for the COVID-19 virus. *World Heal. Organ.* 1–9 (2020).

14. Yoshino, N., Taghizadeh-Hesary, F. & Otsuka, M. Covid-19 and Optimal Portfolio Selection for Investment in Sustainable Development Goals. *Financ. Res. Lett.* **38**, 101695 (2021).

15. Arif, M., Hasan, M., Alawi, S. M. & Naeem, M. A. COVID-19 and time-frequency connectedness between green and conventional financial markets. *Glob. Financ. J.* 100650 (2021) doi:10.1016/j.gfj.2021.100650.

16. Qiu, S. (Charles), Jiang, J., Liu, X., Chen, M. H. & Yuan, X. Can corporate social responsibility protect firm value during the COVID-19 pandemic? *Int. J. Hosp. Manag.* **93**, 102759 (2021).

17. Wb, U. G. *et al.* Social Protection and Jobs Responses to COVID-19 : A Real-Time Review of Country Measures. **10**, (2020).

18. Tian, H. *et al.* An investigation of transmission control measures during the first 50 days of the COVID-19 epidemic in China. *Science (80-. ).* **368**, 638–642 (2020).

19. Chen, S., Yang, J., Yang, W., Wang, C. & Bärnighausen, T. COVID-19 control in China during mass population movements at New Year. *Lancet* **395**, 764–766 (2020).

20. McCartney, G. The impact of the coronavirus outbreak on Macao. From tourism lockdown to tourism recovery. *Curr. Issues Tour.* 1–10 (2020).

21. Briz-Redón, Á., Belenguer-Sapiña, C. & Serrano-Aroca, Á. Changes in air pollution during COVID-19 lockdown in Spain: A multi-city study. *J. Environ. Sci. (China)* **101**, 16–26 (2021).

22. Pata, U. K. How is COVID-19 affecting environmental pollution in US cities? Evidence from asymmetric Fourier causality test. *Air Qual. Atmos. Heal.* **13**, 1149–1155 (2020).

23. Venter, Z., Barton, D., Gundersen, V., Figari, H. & Nowell, M. Urban nature in a time of crisis: recreational use of green space increases during the COVID-19 outbreak in Oslo, Norway. *Environ. Res. Lett.* (2020) doi:10.1088/1748-9326/abb396.

24. Day, B. H. The Value of Greenspace Under Pandemic Lockdown. *Environ. Resour. Econ.* **76**, 1161–1185 (2020).

25. Helm, D. The Environmental Impacts of the Coronavirus. *Environ. Resour. Econ.* **76**, 21–38 (2020).

26. Cohen, M. J. Does the COVID-19 outbreak mark the onset of a sustainable consumption transition? *Sustain. Sci. Pract. Policy* **16**, 1–3 (2020).

27. Watts, J. & Kommenda, N. Coronavirus pandemic leading to huge drop in air pollution. *Guard.* **23**, (2020).

28. Mandal, I. & Pal, S. COVID-19 pandemic persuaded lockdown effects on environment over stone quarrying and crushing areas. *Sci. Total Environ.* **732**, 139281 (2020).

29. Bennett, N. J. *et al.* The COVID-19 Pandemic, Small-Scale Fisheries and Coastal Fishing Communities. *Coast. Manag.* **48**, 336–347 (2020).

30. March, D., Metcalfe, K., Tintoré, J. & Godley, B. Tracking the global reduction of marine traffic during the COVID-19 pandemic. (2020).

31. Le Quéré, C. *et al.* Temporary reduction in daily global CO2 emissions during the COVID-19 forced confinement. *Nat. Clim. Chang.* **10**, 647–653 (2020).

32. Newsome, D. The collapse of tourism and its impact on wildlife tourism destinations. *J. Tour. Futur.* (2020) doi:10.1108/JTF-04-2020-0053.

33. Manenti, R. *et al.* The good, the bad and the ugly of COVID-19 lockdown effects on wildlife conservation: Insights from the first European locked down country. *Biol. Conserv.* **249**, (2020).

34. Dowd, J. B. *et al.* Demographic science aids in understanding the spread and fatality rates of COVID-19. *Proc. Natl. Acad. Sci. U. S. A.* **117**, 9696–9698 (2020).

35. Hale, T. *et al.* Variation in government responses to COVID-19 | Blavatnik School of Government. *Work. Pap.* Version 7 (2020).
